# Chemical Composition and Antioxidant Properties of Pecan Shell Water Extracts

**DOI:** 10.3390/antiox11061127

**Published:** 2022-06-08

**Authors:** Nurhan Turgut Dunford, Zinar Pinar Gumus, Canan Sevimli Gur

**Affiliations:** 1 Department of Biosystems and Agricultural Engineering, Oklahoma State University, Stillwater, OK 74078, USA; 2 Robert M. Kerr Food and Agricultural Products Center, Oklahoma State University, Stillwater, OK 74078, USA; 3 Central Research Test and Analysis Laboratory Application and Research Center (EGE-MATAL), Ege University, Izmir 35100, Turkey; zinar.pinar.gumus@ege.edu.tr; 4 Department of Basic Pharmaceutical Sciences, Katip Celebi University, Izmir 35000, Turkey; canan.sevimli.gur@ikc.edu.tr

**Keywords:** pecan shells, antioxidants, extraction, phenolics, byproducts

## Abstract

This study examined the chemical composition and antioxidant properties of the extracts obtained from two byproduct streams generated at a commercial pecan nut shelling operation. Byproduct stream F contained more pecan nut meat pieces and packing material than stream S, consisting of mainly hard outer shell pieces. Samples from Native variety nuts were processed using subcritical, sonication aided and microwave heating, using water as a solvent. Ferric reducing capacity (FRAP), Total Phenolic Content (TPC), 2,2-diphenyl-1-picrylhydrazyl (DPPH), and ABTS [2,2′-azinobis-(3-ethylbenzothiazoline-6-sulfonic acid)] assays were used to determine antioxidant properties of the extracts. The experimental results clearly demonstrated that the chemical composition of the industrial byproducts was significantly different from the hand-separated shells. All the water extracts exhibited significant DPPH, ABTS and FRAP activity. The highest antioxidant capacity was obtained with the extracts obtained via subcritical water at 80 °C. This is the first report published in the literature on the antioxidant properties of water extracts obtained from industrial byproducts from a pecan nut shelling operation processing Native variety. New data generated in this study expand our knowledge of the properties of industrial nut shelling industry byproducts and help to evaluate the potential use of the shell extracts as antioxidants in various applications.

## 1. Introduction

Tree nuts have been a very important part of the human diet for centuries. The US Food and Drug Administration (FDA) acknowledges that “Scientific evidence suggests but does not prove that eating 1.5 ounces per day of most nuts, (such as almonds, Brazil nuts, cashew nuts, hazelnuts, macadamia nuts, pecans, pine nuts, pistachio nuts, and walnuts) part of a diet low in saturated fat and cholesterol may reduce the risk of heart disease” [1]. US Dietary guidelines also emphasize the importance of nut intake for maintenance of good health because of the high concentration of proteins and other health-beneficial nutrients present in nuts [2].

Edible tree nuts such as almond, walnut, hazelnut, Brazil nut, cashew, macadamia and pecan are dry fruits with one seed enclosed in a hard shell. Nuts are shelled to separate edible seed, also referred to as nut meat, from inedible parts. Large amounts of shell produced during nut processing are generally used in low-value applications such as gardening aid, mulch for soil amendment and heating. Considering that shells from some of the tree nuts are rich in phytochemicals possessing antioxidant properties, it is imperative that value-added processing for the recovery of biologically active compounds from industry byproducts be explored.

This article evaluates pecan nut shells as a source of antioxidants. Pecan (*Carya illinoinensis*) is a tree nut native to the US and cultivated in southern states including Oklahoma, Texas and Louisiana. A number of studies reported the presence of health-beneficial phytochemicals in pecan shells [3,4,5]. Higher levels of phenols and tannins were found in the shells than in the edible part of pecans [3]. The latter compounds have nutritional and medicinal value due to their potent antioxidant properties. Edible nut meat, also referred to as pecan halves, is separated from inedible fractions during the commercial shelling process. Large amounts of shells produced during the latter process represent about 40% to 50% of the whole nut weight. Therefore, pecan nut shelling industry byproducts have economic significance and potential for valorization. Most of the studies on the characterization of pecan shell extracts were carried out using hand-separated pecan shells rather than evaluating actual byproducts from commercial operations [3,4,5]. Indeed, a recent study performed in our research group [5] addressed the issue and evaluated the biological activity of aqueous ethanol extracts obtained directly from industrial byproducts. Considering that hand-separated nut shells would have a significantly different composition than that of the industrial byproducts and a process based on hand-separated shells would not be commercially viable, we continue to investigate potential valorization options for industrial pecan processing byproducts.

It has been reported that the Oxygen Radical Absorbance Capacity (ORAC), ferric reducing capacity (FRAP) and Total Phenolic Content (TPC) of acetone extracts obtained from eighteen pecan cultivars grown in Georgia, New Mexico and Texas were all significantly different [6]. The antioxidant capacity of the extracts increased as the phenolic content increased in the samples. The largest contributors to the high antioxidant capacity were gallic acid, catechin, and ellagic acid [6]. The efficacy of the pecan nut shell extracts for providing protection against oxidative damage induced by cyclophosphamide in different organs was demonstrated in animal models [7]. It appears that pecan shell extracts also minimize rat liver damage triggered by oxidative stress caused by chronic ethanol intake [8]. Flavonol methyl ether: caryatin-3′ sulfate and caryatin-3′ methyl ether-7-O-β-d-glucoside isolated from pecan nut shell have shown significant antioxidant activities in diabetic rats [9].

It is important to note that variety, growth location, climate and agronomic practices significantly affect the chemical composition of plants [4,5]. Furthermore, the extraction method and solvent type used in the process are the key parameters determining the properties of the extracts obtained from nut shells. The current study examines the antioxidant properties of the water extracts obtained from the byproducts generated at industrial shelling facilities processing pecan nuts grown in Oklahoma. The effects of the extraction method on the chemical composition of the samples and correlations between chemical composition and antioxidant properties are also investigated. Antioxidant capacity data generated in this study are compared to the data previously published in the literature. This study is novel because it contributes new scientific data on the antioxidant properties of pecan shell extracts obtained from industrial byproducts and discusses challenges and opportunities for the valorization of the industrial byproducts.

## 2. Materials and Methods

### 2.1. Materials

#### 2.1.1. Pecan Shell Samples

**Figure 1:**
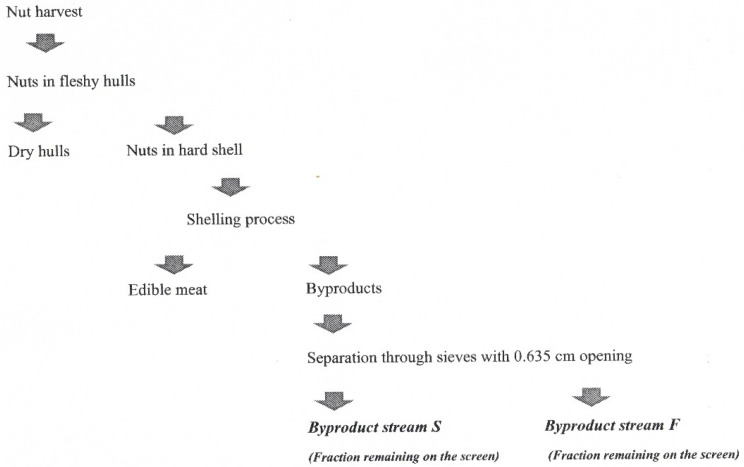
Pecan nut processing flow chart.

Native variety pecans grown in Bristow, Oklahoma, USA, are examined in this study. Pecan samples harvested and shelled in 2018 were received directly from a facility operating in Oklahoma. The commercial pecan shelling process was described elsewhere [5,10]. Two types of pecan shell samples labeled S and F were used in the experiments. The labels S and F represent materials remaining on screens with 0.635 cm (1/4 in.) openings and the finer particles passing through the same screens, respectively (Figure 1). The particle size of the two byproducts, S and F, were further reduced by grinding using a hammer mill (Fitz Mill DAS06, The Fitzpatrick Company, Westwood, MA, USA), followed by a second grinding with a coffee grinder (Mr. Coffee W183ME, Newell Brands, Atlanta, GA, USA). The ground samples were placed in plastic Ziploc bags and kept at −20 °C until further analysis.

#### 2.1.2. Reagents

All the chemicals including 2,2-diphenyl-1-picrylhydrazyl (DPPH) (over 95% purity), Folin–Ciocalteu reagent, H_3_PO_4_ (HPLC grade), methanol (HPLC grade), acetonitrile (HPLC grade), FeCl_3_ (over 97% purity), HCl (HPLC grade), Trolox (over 98% purity), TPTZ (over 95% purity) were purchased from Sigma-Aldrich, Inc., St. Louis, MO, USA.

### 2.2. Methods

#### 2.2.1. Proximate Composition of Pecan Shells

A carbon-nitrogen analyzer (Leco TruSpec CN, St. Joseph, MI, USA) was used to determine the protein content of the samples [11]. The moisture, ash, and lipid contents of the samples were analyzed according to the American Association of Cereal Chemists (AACC) Method 44-15A [12], Association of Official Analytical Chemists (AOAC) Method 923.03, and AOAC Method 960.39 [11,13], respectively.

#### 2.2.2. Extraction

The effect of the extraction method on extract chemical composition and its antioxidant capacity was examined by comparing the water extracts obtained by three different methods; extractions with subcritical water, sonication aided and microwave heating. Subcritical water extraction was carried out using a Dionex ASE 350 model (Thermo Scientific, Waltham, MA, USA) unit at four different temperatures, 80, 100, 125 and 150 °C, with 100% deionized water, static time 5 min, 3 cycles, flush 100%, purge 60 s at 1500 psi. Sonication aided extraction was performed by treating the ground pecan shell samples at sonication amplitude of 5 (500 V (rms)/20 kHz) for 1 h (Sonic Dismembrator, Model 550, Fisher Scientific, Pittsburg, PA, USA). An adjustable power 1.55 kW microwave oven was also used for pecan shell extraction. The microwave power setting was 10 (maximum power). The deionized water/ground sample (10 g) ratio was 20/1 on a weight basis for both sonication aided and microwave extraction experiments. The extracts were freeze-dried and stored at −20 °C away from light until further analysis.

#### 2.2.3. Chemical Composition of Shell Extracts

Chromatographic separation of the compounds present in pecan shell extracts was carried out using an HPLC system (1260 Infinity series, Agilent Technologies, Santa Clara, CA, USA) equipped with a pump, an online degasser, an auto sampler, and a Diode Array Detector (DAD). Elution of the peaks in the chromatogram was performed with a C18 reverse-phase column (4.6 mm × 25 cm), type Spherisorb ODS-2 5 μm, 100 A° (Waters Corporation, Milford, MA, USA). The detector wavelength was set at 280 nm. Sample injection volume was 20.0 µL. The column temperature was maintained at 35 °C. A ternary linear elution gradient consisting of water with 0.2% H_3_PO_4_ (*v*/*v*) (A), methanol (B) and acetonitrile (C) was used at a flow rate of 1.0 mL/min. Separation of the chemical species was achieved using the elution method shown in Table 1. Freeze-dried samples were dissolved in 80/20 methanol/water (*v*/*v*) solution for HPLC injection. The analytical merits of the HPLC method are summarized in Table 2.

#### 2.2.4. Antioxidant Capacity Tests

##### DPPH Radical Scavenging Capacity

The 2,2-diphenyl-1-picrylhydrazyl (DPPH) free radical scavenging capacity of the extracts was determined as follows; absorbance of the 2 mL of 60 μM DPPH solution + 50 μL of the extract was recorded using a spectrophotometer (Beckman DU520 UV/Vis, Beckman Coulter, Inc., Brea, CA, USA) at 517 nm for 60 min at 30 s intervals. The following equation was used to calculate the radical inhibition capacity of the sample:

$$\mathrm{DPPH}\;\mathrm{inhibition}\;\left(\%\right)=\left[\frac{\left(A_{control}-A_{sample}\right)}{A_{control}}\right]\times 100$$

where: *A_sample_* = absorbance of the sample at 60 min, *A_control_* = absorbance of the assay solution at 0 min.

##### Total Phenolic Content

A mixture of 0.1 mL of extract, 500 μL of Folin-Ciocalteu reagent, 1.5 mL of 20% sodium carbonate, and 7.9 of mL deionized water was allowed to react at ambient temperature (22 °C) for 2 h. Then, the absorbance of the mixture was measured at 765 nm using a spectrophotometer (Beckman DU520 UV-VIS, Beckman Coulter, Inc., Brea, CA, USA). A standard curve prepared with gallic acid (Sigma-Aldrich, St. Louis, MO, USA) was used to quantify the absorbance readings. Total phenolic contents (TPC) of the samples were reported as gallic acid equivalent (GE) per gram of solid sample used for extraction.

##### Ferric Reducing Capacity (FRAP)

The FRAP reagent was prepared mixing acetate buffer (0.3 M, pH 3.6), FeCl_3_ (20 mM), TPTZ (10 mM) and HCl (40 mM) in proportions of 10:1:1 (*v*/*v*/*v*), respectively. Extract or Trolox (50 μL) was mixed with 3 mL of freshly prepared FRAP reagent and the mixture was incubated at 37 °C for 30 min. Then, the absorbance of the mixture was recorded at 593 nm. A Trolox standard curve prepared according to the FRAP assay used for the extracts was employed to express the assay results as Trolox equivalent (TE). The FRAP values were expressed as μmol TE/g of pecan shell.

##### ABTS Assay

An ABTS [2,2′-azinobis-(3-ethylbenzothiazoline-6-sulfonic acid)] assay was also used to evaluate the antioxidant potential of pecan shell extracts. The test protocol was based on the ABTS assay described by Re et al. [14]. In summary, the ABTS^+^ solution was prepared by mixing an equal volume of ABTS^−2^ stock solution (7.4 mM in water) with potassium persulfate (2.6 mM in water) and incubating the mixture for 12 h in dark. The extract, 150 µL, was mixed with 2850 µL ABTS^+^ solution which was diluted with methanol to an absorbance value of 1.1 at 734 nm. Then, the absorbance of the extract and ABTS^+^ mixture was recorded at 734 nm after 30 s of mixing. A Trolox standard curve which was prepared according to the ABTS assay used for the extracts was employed to express the assay results as TE.

### 2.3. Statistical Analyses

All analytical tests for sample characterization were carried out at least in duplicate. Means were compared using the least significance difference (Tukey’s HSD test, *p* > 0.05) method. The analysis of variance (ANOVA) of the experimental data was performed using SAS/STAT (ver. 9.3, SAS Institute Inc., Cary, NC, USA). All statistical tests were performed at the *p* = 0.05 level of significance.

### 2.4. Principal Component Analysis (PCA)

PCA was performed using MINITAB 15 Statistical Software (Minitab, LLC, State College, PA, USA). Similarities and differences between main groups and observations were presented as score plots. The loading plots have been used to explain the relationship between variables in the score plots and cluster observations.

## 3. Results and Discussion

### 3.1. Chemical Composition of Byproduct Streams

In a previous publication, we reported the anticancer and antioxidant properties of aqueous ethanol extracts obtained from the shell of the pecans grown and processed in Oklahoma, USA [5]. The current study examined the effect of the extraction method on the antioxidant properties of pure water extracts. This study is also different from the previous studies (Table 3) because the experiments were carried out using two different byproducts streams generated at commercial shelling operations rather than the shells hand separated in a laboratory. Considering that the characteristics of hand-separated materials would be very different from hand-separated shells, data on the industrial byproducts are crucial for evaluating the potential of pecan shell valorization at a commercial scale.

Indeed, Table 4 clearly demonstrates that the proximate composition of the industrial byproducts (as is) is significantly different than that of the hand-separated (cleaned) samples. Industrial byproducts contained a substantial amount of oil. Hand separation of the large nut meat pieces from the samples resulted in a significant decrease in the oil content of the samples (Table 4). The latter result can be explained by the high lipid content of pecan nut meat, over 65% [4]. The efficiency of the mechanical separation process used by the industry varies depending on the processing conditions and pecan variety. During the shelling process, pecan halves or meat are broken down into a broad range of particle sizes [10]. Broken meat pieces end up in the byproducts. It is also important to note the significant difference in oil content in the byproduct streams S and F, which is due to the physical properties of the nut components. The packing tissue present between the shell and meat and the middle septum separating pecan halves inside the nut is very brittle. Broken meat pieces are softer than the hard-outer shell and stick to the packing tissue. They fragment into finer particles than shell pieces. Hence, the sample from the S stream contains mostly hard-shell pieces which are low in oil and protein contents while stream F contains more meat pieces resulting in higher oil content than that in the S stream. Indeed, our previous study with the same pecan shelling operation byproducts demonstrated that fatty acids commonly found in pecan nut oil were among the major chemical compounds identified in the extracts obtained from the byproducts [5].

This study focused on the Native variety grown and processed in Oklahoma. The reason for choosing the Native variety for the study is that data on the chemical composition of the extracts obtained from this variety are very limited. To the best of our knowledge, there is only one study examining aqueous ethanol extracts from the Native variety [5]. The data presented in this publication are new and help us better understand the chemical characteristics of the pecan nut shelling industry byproducts.

### 3.2. Antioxidant Properties of Pecan Nut Shell Extracts

The antioxidant properties of various plant extracts are commonly attributed to their phenolic content [22]. There were significant differences in the TPC content of the water extracts obtained with different extraction methods, byproduct streams and temperatures (Table 5). In general, extracts obtained from byproduct stream F had higher TPC content than that from stream S. The latter result could be partly due to the higher oil content in stream F, and consequently, in the extracts. In a previous study, we have shown that extract yield from the F stream is significantly higher than that from the S stream due to the differences in oil content of the samples [5]. It is well established that pecan nut oil contains a significant amount of tocopherols, 88–420 mg/kg, which contribute to the TPC content in the extracts [23].

There was a strong negative correlation between the ASE extraction temperature and TPC content of the extracts, specifically for the extracts from stream F, (correlation equation: y = −2.5467x + 649.38 with R² = 0.9649). As the temperature increased, the TPC content of the extracts from stream F decreased significantly (see the negative slope of the correlation equation in the previous statement). Yet, the temperature and TPC correlation for the extracts from stream S was very weak with a small slope (y = −0.1796x + 291.46, R² = 0.0332). Considering that the oil content in S is significantly lower than that in F, the effect of the oil amount and its tocopherol content in the extracts from stream S is expected to be relatively low. At the highest temperature examined in this study, 150 °C, the difference between TPC contents of F and S extracts is relatively low, probably because of the higher oil content and its higher concentration of non-phenolic components (i.e., fatty acids) in the F extracts diluting the TPC contribution from tocopherols in the oil. It appears that sonication aided extraction of the S sample was beneficial for increasing the TPC content of the extracts. The TPC content of the microwave extracts from F was within the range obtained with the ASE method.

Antioxidant assays can be categorized as hydrogen atom transfer (HAT) or single electron transfer (ET) based assays. In a HAT-based method, free radicals can react with both antioxidants and the substrates present in the system. The antioxidant donates its hydrogen atom to the free radical faster than the substrate, thus retarding the oxidation of the substrate. The formation of stable radicals slows down or inhibits the oxidation chain reactions. Therefore, HAT-based assays follow a competitive reaction kinetic. ET-based assays have only two components, antioxidants and oxidants, with no substrate. In ET-based assays, the oxidant is used as an indicator to monitor the reaction [24] and measure the capacity of an antioxidant in the reduction of an oxidant [24,25,26,27]. Trolox equivalence antioxidant capacity (TEAC), FRAP, “total antioxidant potential” assay using a Cu (II) complex as an oxidant, and DPPHT are ET-based tests.

DPPH which is a stable organic nitrogen free radical is often used to evaluate the antioxidant capacity of plant extracts. This assay measures the reducing capacity of antioxidants toward DPPH. This study demonstrated that there were significant differences in the DPPH reducing capacity of the extracts obtained from two different byproduct streams by various extraction methods (Table 5). The lowest and highest DDPH inhibition values were found in the extracts obtained with the ASE method at 150 °C using byproduct stream S and at 80 °C with byproduct stream F, respectively. For the ASE method, the effects of temperature, byproduct type and temperature * byproduct type interaction on DPPH were significant, *p* < 0.01. Pearson correlation coefficient for DPPH vs. TPC was quite low, 0.566.

The good spectral characteristics and solubility of ABTS in both organic and aqueous media and its stability in a wide pH range make this assay an advantageous option for the estimation of the antioxidant activity of plant extracts [28]. There are reports indicating that ABTS radicals are scavenged through hydrogen atom donation [29], as well as through electron transfer [30] or even with a combination of the two mechanisms [31]. The extract with the highest ABTS value was obtained with the sample obtained via ASE extraction at 80 °C using byproduct stream S. The effects of temperature, byproduct type and temperature * byproduct type interaction on ABTS were significant, *p* < 0.01, for the ASE extracts. The lowest ABTS value was found with the extract obtained by microwave heating using stream S. The Pearson coefficient for ABTS vs. TPC was low, 0.425.

FRAP assay is based on the single-electron transfer mechanism in which antioxidant is oxidized by oxidants, in this case, Fe(III), and as a result, an electron is transferred from antioxidant to oxidant [32]. There were significant differences among the FRAP values for the samples treated differently. However, no apparent trend could be established by extraction method, temperature or byproduct type (Table 5). The highest FRAP value was recorded for the extract obtained with the ASE method at 100 °C. The lowest FRAP value was for the sample extracted from byproduct stream S using microwave heating. The effects of temperature, byproduct type and temperature * byproduct type interaction on FRAP values were significant, *p* < 0.01, for the ASE extracts. The Pearson coefficient for the FRAP vs TPC was similar to those obtained with DPPH and ABTS assays.

Significant differences in antioxidant activity of the samples by processing conditions and the assay used for evaluation with no apparent trend certainly emphasize the complexity of natural product extract compositions and resulting synergistic and/or antagonistic interactions of the compounds present in the samples.

### 3.3. Composition of Phenolic Compounds in Pecan Nut Shell Extracts

The effect of phenolic composition of the pecan shell extracts on their antioxidant activity was examined (Table 6). 

**Figure 2:**
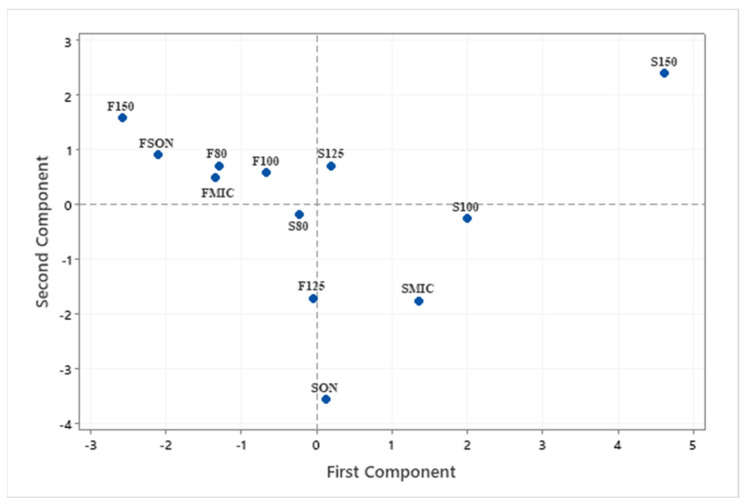
Score plot highlighting effect of extraction method on extract composition. Effect of the phenolic compounds on the components.

**Figure 3:**
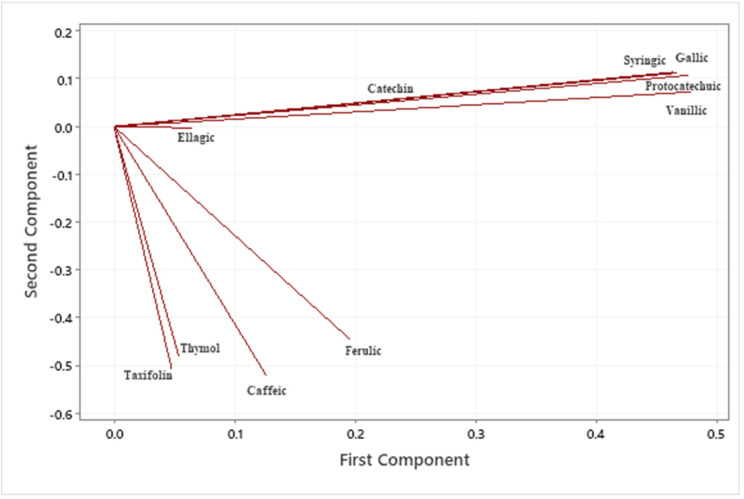
Loading plot.

**Figure 4:**
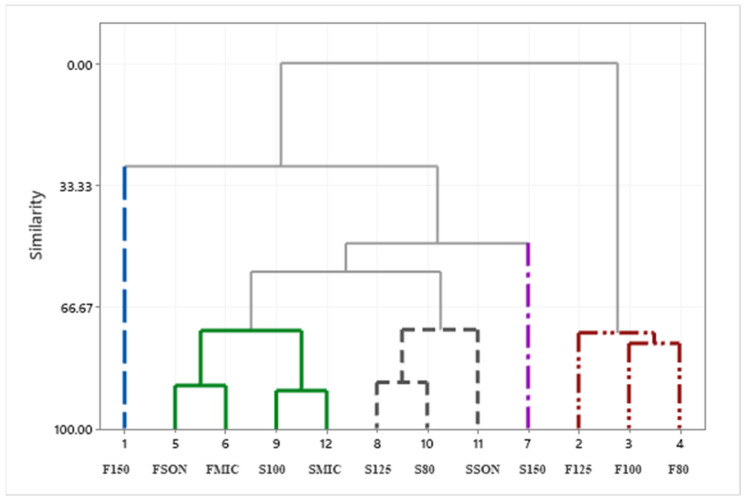
Cluster analyses of the pecan shell extracts.

Gallic, protocatechuic, vanillic, caffeic, syringic, elagic acids were the major compounds present in all the extracts examined in this study. Taxifolin and thymol were the other phenolic compounds found in some extracts, but not all. Phenolic compound with the highest concentration was elagic acid, 96.0 µg/mL, in the extract obtained by the ASE method at 100 °C using the byproduct stream F. Considering that extract composition is significantly affected by several variables; extraction method, byproduct type and temperature, the compositional data were evaluated by a statistical analysis method known as Principal Component Analysis which is used to identify a smaller number of uncorrelated variables, called “principal components”, from a large set of data. The data scales on Figure 2 and Figure 3 were calculated using the original experimental data. Eigenanalysis of the correlation matrix showed that the first principal component (Eigenvalue 3.8500) accounts for 38.5% of the total variance. The score plot (Figure 2) which displays the clusters, trends, and outliers in the first two principal components, revealed a broad scattering, ASE extracts at 150 °C with both byproducts stream, F150 and S150, located at each end of the spectrum. As seen in the loading plot (Figure 3), gallic (0.465), protocatechuic (0.475), vanillic (0.477) and syringic (0.463) acids were the compounds that correlated the most with the first principal component. All the compounds identified in the samples were positively correlated with the first principal component. The second component had large negative correlations with ferulic (−0.444), caffeic (−0.519), thymol (−0.479) and taxifolin (−0.509) (Figure 3 and Table 5). Similarities and differences in the phenolic composition of the samples were further analyzed via Cluster Analysis of the observations (Figure 4). When the number of the clusters was set at 5 (similarity level 57.6% and Euclidean distance at 25.4033) samples FSON, FMIC, S100, SMIC were in the group 1, S125, S80 and SSON formed the group 2, F125, 100 and F80 group 3 and F150 and S150 were in groups 4 and 5, respectively. Although there was no apparent trend by the extraction method or byproduct type at the low cluster numbers, the dendrogram (Figure 4) indicates higher similarity and lower distance by byproduct stream at a higher number of clusters. The latter statistical analyses clearly demonstrate the complex interactions of extraction method, temperature and byproduct stream on extract phenolic composition. The weak correlations between the concentration of the individual phenolic compounds in the extracts and their antioxidant properties (Table 7) further emphasize the major interactions among processing parameters, extract composition and antioxidant properties. Considering that this study focuses on the characterization of the crude pecan shell extracts, the lack of a clear trend by extraction method and byproduct type is not surprising.

## 4. Conclusions

This study established that proximate compositions of pecan shells generated at commercial nut shelling operations were significantly different than those shell samples hand separated in the laboratory. Extracts from the byproduct stream F contained significantly higher oil content than that from the S stream. In general, TPC contents of the water extracts from the byproduct stream F were higher than those of extracts from byproduct stream S. No clear trend in the antioxidant properties of the water extracts could be established with extraction method, byproduct stream or phenolic content of the extracts. However, it appears that ASE extraction of byproduct stream F at 80 °C is the optimal process for obtaining a water extract with high DPPH, TPC, ABTS and FRAP activities. Considering that this study examined the crude nut shell extracts, weak correlations between process variables and antioxidant properties of the samples were expected. The latter results emphasize the complexity of the extract compositions and significant interactions between process variables and byproduct streams. Experimental data presented in this study are valuable for a better understanding of the potential valorization of shell extracts as antioxidants to be used in various applications. Further research on downstream processing and purification of crude extracts could enhance the antioxidant properties of nut shell extracts and allow the custom formulation of extract composition for specific applications.

## Figures and Tables

**Table 1:** HPLC mobile phase gradient *.

Time (min)	A (%)	B (%)	C (%)
0	96	2	2
40	50	25	25
45	40	30	30
50	0	50	50
52	0	50	50
55	96	2	2

* (A) water with 0.2% H3PO4 (*v*/*v*), (B) methanol and (C) acetonitrile.

**Table 2:** Analytical merits of the HPLC method [limit of detection (LOD), limit of quantification (LOQ), calibration equation (CE), and square of correlation coefficient (R^2^)].

Compound	Retention Time (min)	CE	R^2^	LOD (µg/mL)	LOQ (µg/mL)
Ferulic Acid	28.574	y = 13.899x − 2.0306	0.9999	0.27	0.90
Vanilic Acid	21.167	y = 32.748x − 0.8276	0.9999	0.15	0.49
Ellagic Acid	31.205	y = 10.283x − 2.2246	0.9999	0.20	0.66
Gallic Acid	9.146	y = 51.982x − 11.556	0.9999	0.17	0.57
Caffeic Acid	21.873	y = 17.314x + 2.1885	0.9999	0.27	0.92
Thymol	51.379	y = 13.732x + 1.3354	0.9997	0.25	0.83
Taxifolin	29.443	y = 37.837x − 13.664	0.9998	0.17	0.56
Catechin	18.603	y = 9.4667x + 1.9891	0.9999	0.27	0.91
Syringic Acid	22.102	y = 55.622x + 8.7233	0.9999	0.11	0.35
p-coumaric acid	27.073	y = 54.957x + 6.2524	0.9999	0.17	0.58
Protocatechuic acid	14.074	y = 24.798x − 1.9328	0.9998	0.20	0.66

**Table 3:** Effect of extraction method on antioxidant capacity of pecan shell extracts.

Extraction/Solvent	Water	Aqueous Ethanol	Reference
TPC (mg GAE/g extract)	37.3–444.1	153.5–581.9	[3,5,7,8,15,16,17,18,19,20,21]
DPPH (% inhibition)	46.1–86.8	79.4–90.8	[19], This work
DPPH (Trolox Equivalent, μmol/g extract)	346.6–1268.0	524.8–1287.1	[15,16,20]
ABTS (Trolox Equivalent, μmol/g extract)	368.3–5390.4	1562.5–2573.0	[3,7,18,19] This work
FRAP (Trolox Equivalent, μmol/g extract)	4267.2–5800.2	-	This work

**Table 4:** Proximate composition (weight %) of pecan shelling industry byproducts as received from the processing facility and hand separation of nut meat pieces *.

Sample	Oil (as Is)	Oil (Cleaned)	Protein (as is)	Protein (Cleaned)	Ash (as Is)	Ash (Cleaned)	Moisture (as Is)	Moisture (Cleaned)
Native-F (Bristow, OK)	12.9 ± 0.4 ^a^	7.1 ± 0.4 ^a^	3.5 ± 0.2 ^a^	2.4 ± 0.3 ^a^	1.3 ± 0.07 ^a^	1.5 ± 0.04 ^a^	16.01 ± 0.06 ^a^	15.13 ± 0.06 ^a^
Native-S (Bristow, OK)	2.8 ± 0.1 ^b^	0.72 ± 0.07 ^b^	1.9 ± 0.1 ^b^	1.81 ± 0.01 ^b^	1.73 ± 0.02 ^b^	1.801 ± 0.007 ^b^	12.82 ± 0.03 ^b^	13.1 ± 0.2 ^b^

* Data are presented as mean ± standard deviation. Means in the same column with the same letter are not significantly different (Tukey’s HSD test, *p* > 0.05).

**Table 5:** Antioxidant potential of pecan shell extracts *.

Sample	DPPH (% Inhibition)	TPC (mg GAE/g Extract)	ABTS (Trolox Equivalent, μmol/g Extract)	FRAP (Trolox Equivalent, μmol/g Extract)
F_150_	46.1 ± 0.3 ^f^	259.8 ± 2.7 ^h^	4920.6 ± 0.9 ^i^	4494.0 ± 3.8 ^j^
F_125_	76.9 ± 1.3 ^cd^	349.0 ± 3.2 ^c^	5205.0 ± 3.5 ^e^	5217.6 ± 1.9 ^d^
F_100_	81.4 ± 1.6 ^abc^	385.5 4.3 ^b^	5345.6 ± 0.9 ^b^	5800.2 ± 5.0 ^a^
F_80_	86.8 ± 0.8 ^a^	444.1 ± 4.3 ^a^	5390.4 ± 2.3 ^a^	5653.0 ± 1.1 ^b^
S_150_	33.7 ± 1.3 ^g^	284.2 ± 3.4 ^fg^	5053.8 ± 1.8 ^g^	5495.2 ± 2.1 ^c^
S_125_	66.7 ± 2.1 ^e^	223.3 ± 4.8 ^i^	5131.9 ± 2.7 ^f^	5018.8 ± 0.1 ^e^
S_100_	76.8 ± 0.4 ^cd^	294.3 ± 4.2 ^f^	5195.6 ± 4.4^e^	4907.0 ± 1.1 ^f^
S_80_	77.6 ± 0.2 ^bcd^	276.8 ± 3.8 ^g^	5253.8 ± 1.8 ^d^	4754.2 ± 2.9 ^h^
F_SON_	82.4 ± 1.1 ^abc^	333.7 ± 3.2 ^d^	5338.1 ± 0.8 ^b^	4880.6 ± 0.9 ^g^
S_SON_	74.1 ± 4.1 ^d^	310.4 ± 0.2 ^e^	5041.2 ± 3.6 ^h^	4267.2 ± 2.2 ^k^
F_MIC_	83.5 ± 0.8 ^ab^	362.1 1.7 ^c^	5288.1 ± 0.9 ^c^	4693.1 ± 4.4 ^i^
S_MIC_	78.3 ± 1.3 ^bcd^	287.8 ± 1.7 ^fg^	4069.5 ± 2.5 ^j^	4198.8 ± 3.5 ^l^

* The sample codes represent byproduct type and extraction method: F150 = ASE extraction of the sample from byproduct stream F at 150 °C; F125 = ASE extraction of the sample from byproduct stream F at 125 °C; F100 = ASE extraction of the sample from byproduct stream F at 100 °C; F80 = ASE extraction of the sample from byproduct stream F at 80 °C; FSON = Sonication aided extraction of the sample from byproduct stream F; FMIC = Microwave extraction of the sample from byproduct stream F; S150 = ASE extraction of the sample from byproduct stream S at 150 °C; S125 = ASE extraction of the sample from byproduct stream S at 125 °C; S100 = ASE extraction of the sample from byproduct stream S at 100 °C; S80 = ASE extraction of the sample from byproduct stream S at 80 °C; SSON = Sonication aided extraction of the sample from byproduct stream S; SMIC = Microwave extraction of the sample from byproduct stream s at 80 °C. Means in the same column with the same letter are not significantly different (Tukey’s HSD test, *p* > 0.05). Data are presented as mean ± standard deviation.

**Table 6:** Effect of extraction method on phenolic content pecan shell extracts *.

Sample	Protocatechuic	Gallic	Catechin	Vanillic	Caffeic	Syringic	Ferulic	Taxifolin	Elagic	Thymol
F150	3.7 ± 0.3 ^f^	1.6 ± 0.6 ^h^	1.5 ± 0.1 ^f^	4.4 ± 0.2 ^e^	2.9 ± 0.3 ^f^	1.6 ± 0.1 ^gh^	8.8 ± 0.2 ^e^	n.d.	6.5 ± 1.9 ^i^	n.d.
F125	3.87 ± 0.02 ^f^	20.3 ± 0.5 ^bc^	25.5 ± 0.3 ^a^	4.4 ± 0.2 ^e^	5.7 ± 0.2 ^b^	0.6 ± 0.1 ^j^	19.1 ± 0.5 ^b^	8.25 ± 0.04 ^b^	87.3 ± 0.1 ^b^	0.15 ± 0.01 ^cd^
F100	7.7 ± 0.1 ^d^	12.6 ± 0.1 ^e^	25.2 ± 0.8 ^a^	4.401 ± 0.001 ^e^	3.5 ± 0.1 ^ef^	1.070 ± 0.001 ^i^	n.d.	4.9 ± 0.2 ^g^	96.0 ± 1.4 ^a^	0.28 ± 0.03 ^bc^
F80	4.5 ± 0.2 ^f^	12.8 ± 0.2 ^e^	21.4 ± 0.5 ^b^	4.1 ± 0.1 ^ef^	4.4 ± 0.1 ^d^	0.490 ± 0.001 ^j^	7.85 ± 0.08 ^e^	5.49 ± 0.02 ^f^	74.7 ± 0.5 ^c^	n.d.
FSON	0.83 ± 0.05 ^h^	9.1± 0.5 ^g^	16.269 ± 0.001 ^cd^	3.998 ± 0.001 ^ef^	3.3 ± 0.2 ^ef^	1.268 ± 0.001 ^hi^	n.d.	6.20 ± 0.02 ^e^	50.5 ± 5.2 ^ef^	n.d.
FMIC	1.72 ± 0.06 ^g^	15.8 ± 0.3 ^d^	21.7 ± 0.6 ^b^	3.8 ± 0.1 ^f^	4.5 ± 0.2 ^cd^	1.786 ± 0.001 ^g^	n.d.	6.4 ± 0.1 ^d^	57.5 ± 1.2 ^de^	n.d.
S150	17.8 ± 0.2 ^a^	41.6 ± 1.1 ^a^	24.8 ± 0.1 ^a^	12.031 ± 0.001 ^a^	3.706 ± 0.001 ^e^	10.7 ± 0.2 ^a^	8.1 ± 0.2 ^e^	2.21 ± 0.01 ^i^	51.8 ± 1.2 ^e^	n.d.
S125	7.92 ± 0.09 ^d^	14.35 ± 0.01 ^de^	17.7 ± 0.4 ^c^	6.6 ± 0.2 ^c^	5.3 ± 0.2 ^b^	4.430 ± 0.001 ^d^	6.21 ± 0.04 ^f^	2.82 ± 0.03 ^h^	43.7 ± 1.0 ^f^	n.d.
S100	10.1 ± 0.5 ^b^	21.9 ± 0.3 ^b^	21.6 ± 0.7 ^b^	7.7 ± 0.1 ^b^	5.3 ± 0.1 ^b^	8.2 ± 0.1 ^b^	14.4 ± 0.3 ^c^	7.61 ± 0.05 ^c^	59.7 ± 0.6 ^d^	n.d.
S80	6.10 ± 0.05 ^e^	10.9 ± 0.1 ^f^	17.4 ± 0.4 ^cd^	6.8 ± 0.2 ^c^	5.274 ± 0.001 ^b^	2.6 ± 0.1 ^f^	13.1 ± 0.6 ^d^	4.98 ± 0.01 ^g^	35.3 ± 0.2 ^g^	n.d.
SSON	6.6 ± 0.2 ^e^	10.51 ± 0.07 ^fg^	11.3 ± 0.2 ^ed^	6.1 ± 0.1 ^d^	7.1 ± 0.3 ^a^	3.010 ± 0.001 ^e^	20.8 ± 0.3 ^a^	9.30 ± 0.01 ^a^	25.6 ± 0.2 ^h^	0.5 ± 0.2 ^a^
SMIC	8.93 ± 0.01 ^c^	19.4 ± 0.3 ^c^	15.6 ± 0.7 ^d^	8.2 ± 0.2 ^b^	5.1 ± 0.1 ^bc^	5.3 ± 0.1 ^c^	15.2 ± 0.4 ^c^	9.16 ± 0.05 ^a^	53.2 ± 0.2d ^e^	0.41 ± 0.01 ^ab^

* See Table 5 for the sample codes. Means in the same column with the same letter are not significantly different (Tukey’s HSD test, *p* > 0.05). Data are presented as mean ± standard deviation.

**Table 7:** Pearson correlation coefficient for individual phenolic compounds and DPPH, ABTS and FRAP activity of the pecan nut shell extracts.

Phenolic Compound	DPPH	ABTS	FRAP
Gallic	0.406	0.025	0.001
Protocatechuic	0.501	−0.016	−0.227
Catechin	0.499	0.350	0.533
Vanillic	0.341	−0.207	−0.452
Caffeic	0.602	0.06	−0.342
Syringic	0.274	−0.104	−0.373
Ferulic	0.329	−0.239	−0.613
Taxifolin	0.347	−0.191	0.020
Thymol	0.361	−0.343	−0.147
Ellagic	0.414	0.255	0.714

## Data Availability

All the data presented in this article are original and obtained via experiments as described in the text.
